# Tirzepatide-induced lichen planus pigmentosus

**DOI:** 10.1016/j.jdcr.2025.02.018

**Published:** 2025-03-10

**Authors:** Craig M. Fisher, Joshua M. Wilberg, Steven T. Rivera, Kevin J. Krauland, Christopher Edens

**Affiliations:** a Department of Dermatology, Wilford Hall Ambulatory Surgical Center, JBSA Lackland, Texas; b Department of Pathology and Area Laboratory Services, Brooke Army Medical Center, Houston, Texas

**Keywords:** GIP, GLP, lichen planus pigmentosus, weight loss

## Introduction

Lichen planus pigmentosus is an acquired pigmentary disorder characterized by hyperpigmented macules in either photodistributed or intertriginous sites. Lichen planus pigmentosus is most commonly seen in Fitzpatrick skin types III-IV and is of unclear etiology.^1^^,^^2^ Cases of drug-induced lichen planus pigmentosus have been reported secondary to infliximab, gefitinib, hydroxyurea, and gold.^3^, ^4^, ^5^, ^6^ Drug-induced cases follow a similar clinical and histopathology pattern as typically lichen planus pigmentosus.

Glucagon-like peptide 1 (GLP-1) receptor and glucose-dependent insulinotropic polypeptide (GIP) receptor agonists have increased in popularity within the recent years both as diabetes and weight loss medications. Tirzepatide is a dual GLP-1 and GIP receptor agonist that is currently utilized for both diabetes and weight loss purposes.^7^ Cutaneous adverse effects to GLP-1 and GIP receptor agonists are uncommon but cases of dermal hypersensitivity reactions, eosinophilic panniculitis, bullous pemphigoid, and morbilliform drug eruptions have been reported.^8^ To our knowledge, no cases of hyperpigmentation or lichen planus pigmentosus have been reported secondary to GLP-1 or GIP receptor agonists.

## Case report

**Fig 1:**
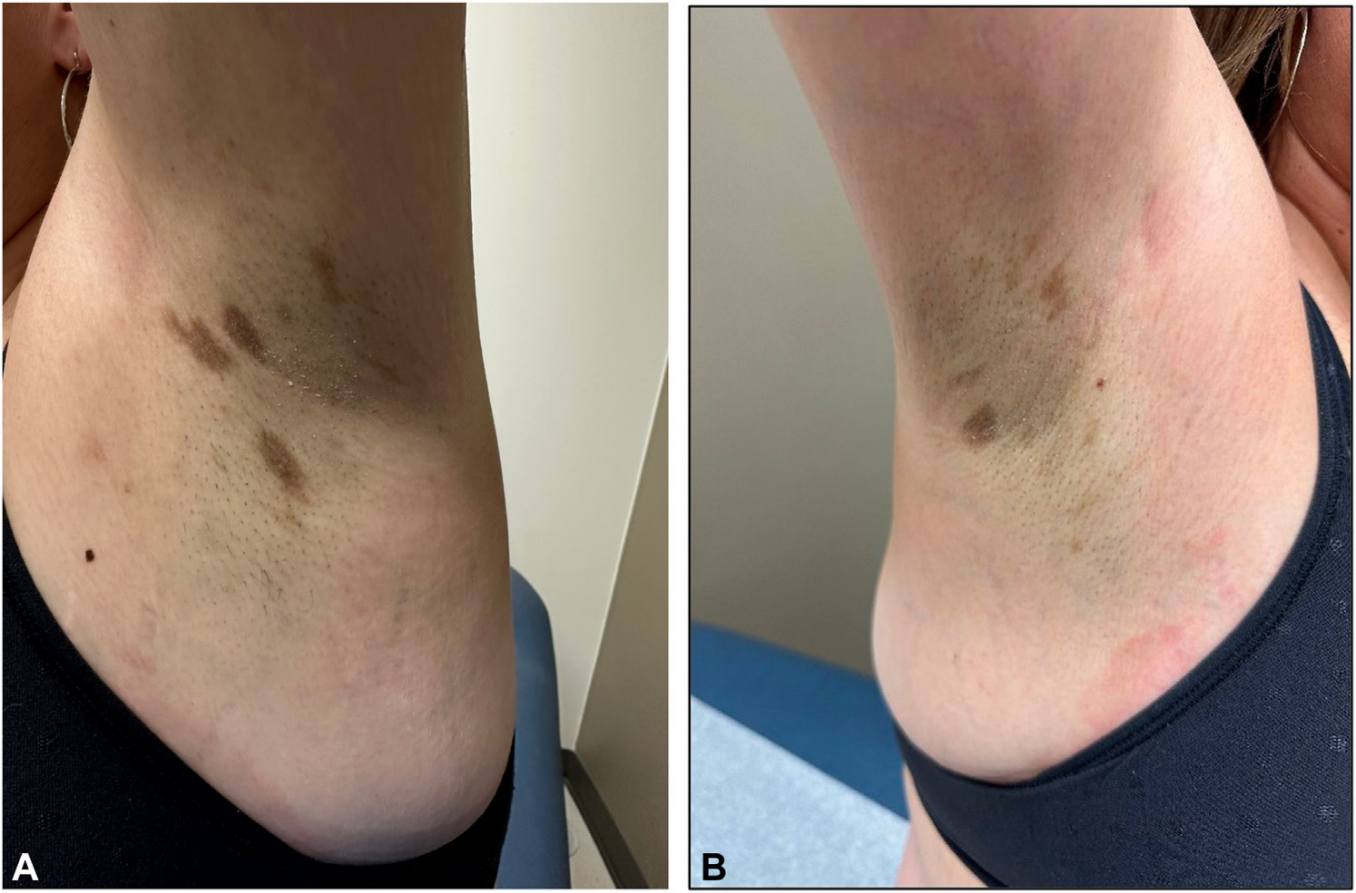
**A** and **B,** Ovoid, well-demarcated, nonerythematous, uniformly brown 6-9 mm macules with scant overlying scale in the bilateral axillae.

**Fig 2:**
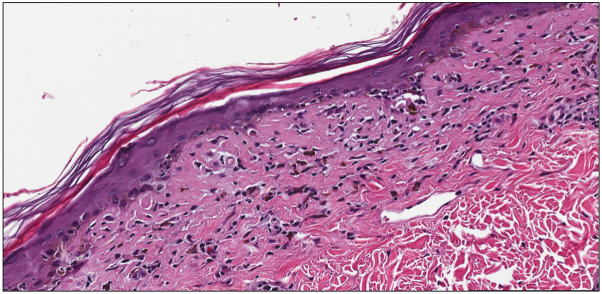
A shave biopsy demonstrated a lichenoid interface dermatitis with dyskeratotic keratinocytes, abundant pigment incontinence, and scattered melanophages in the superficial dermis (hematoxylin and eosin stain, original magnification 20×).

**Fig 3:**
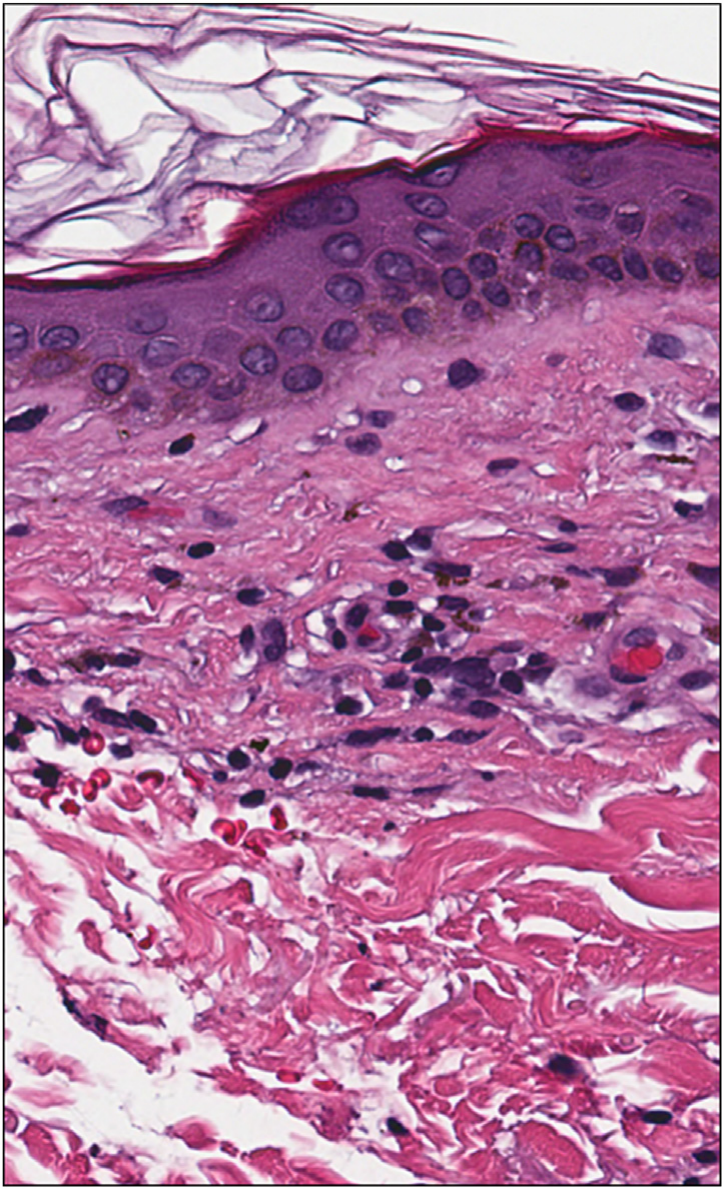
Scattered eosinophils were noted in the lichenoid infiltrate (*arrow*) (hematoxylin and eosin stain, original magnification 40×).

**Fig 4:**
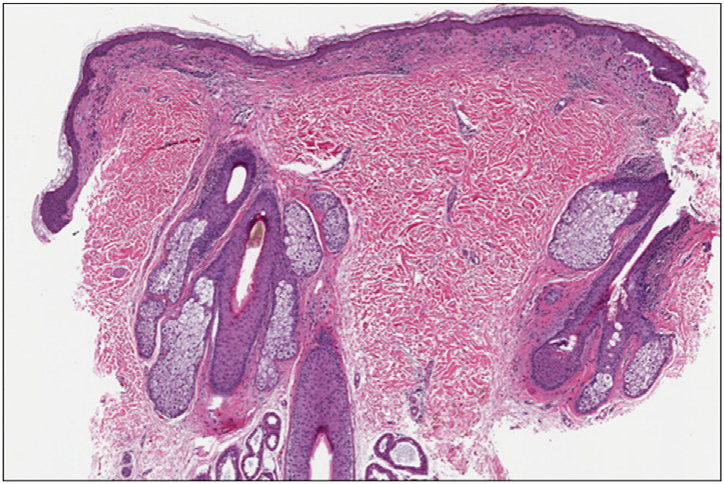
Subtle lichenoid interface dermatitis limited to the superficial dermis without overlying parakeratosis (hematoxylin and eosin stain, original magnification 2×).

A 46-year-old Fitzpatrick skin type II female presented with a 1-year history of pruritic brown macules in the bilateral axillae and groin that began 6-7 months after starting tirzepatide for weight loss. The patient did not report any other new oral, injectable, or topical medications, prescribed or over-the-counter, within the preceding 2 years. She did not report hair, nail, oral, ocular, genital, or systemic symptoms. She indicated the hyperpigmentation and pruritus had remained stable without worsening or improvement for about 10 months. On physical exam, the patient was noted to have well-demarcated nonerythematous uniformly brown macules with scant overlying scale in the bilateral axillae (Fig 1). She reported a history of similar lesions in the groin as well, but no lesions were present at the time of examination. There was no hair loss, scalp erythema or scale, oral mucosal erosions or erythema, ocular lesions, nail changes, or other cutaneous findings on full body skin exam. A shave biopsy was performed which demonstrated a lichenoid interface dermatitis with pigment incontinence (Fig 2, Fig 3, Fig 4). She was started on topical tacrolimus ointment with resolution of associated pruritus but the hyperpigmentation persisted. Additional treatment options to treat the residual hyperpigmentation were discussed but declined by the patient.

## Discussion

The use of medications such as tirzepatide has been on the rise in the treatment of chronic obesity. As the use of the drug increases, additional potential side effects will be noted. This case presents a novel association between tirzepatide and the development of lichen planus pigmentosus, specifically the inversus subtype. Lichen planus pigmentosus is an uncommon, acquired pigmentary disorder that is typically seen in Fitzpatrick skin types III-IV; however, this case occurred in a type II individual. The hyperpigmented macules observed in the intertriginous areas such as the axillae and groin in this case align with the classical presentation of lichen planus pigmentosus.^1^^,^^2^

Previous cases of drug-induced lichen planus pigmentosus have been reported with agents such as infliximab, gefitinib, hydroxyurea, and gold,^3^, ^4^, ^5^, ^6^ but no prior literature describes an association with GLP-1 or GIP receptor agonists such as tirzepatide. This adds to the expanding range of cutaneous adverse effects seen with these newer antidiabetic and weight loss medications, including dermal hypersensitivity reactions and panniculitis.^7^^,^^8^ The mechanism by which tirzepatide induces lichen planus pigmentosus remains unclear, but it could be related to the alteration in immune regulation mediated by GLP-1 and GIP receptor activation.^9^ Given the interface dermatitis seen histologically, immune-mediated injury leading to pigment incontinence and subsequent hyperpigmentation is plausible but will require further investigation.

The patient described in this case had no history of lichenoid dermatoses, other typical risk factors for lichen planus pigmentosus, and did not start any prescription, over-the-counter, or naturopathic medications within the 2 years prior to the onset of the eruption. The average latency period for drug-induced lichenoid eruptions has been reported between 1-208 weeks, with an average of 15.7 weeks, and the majority occurring within the first 12 months.^10^ For our patient, tirzepatide was started within the typical timeline of drug-induced lichenoid eruptions which, in the absence of other risk factors, supports the likely association of tirzepatide with lichen planus pigmentosus in this case. The option of drug discontinuation was discussed with the patient given the lichenoid reaction; however, the patient elected to continue tirzepatide. The risk of development of lichen planus or other lichenoid eruptions following the onset of lichen planus pigmentosus is not well characterized. In this case, the patient was able to receive symptomatic treatment for her pruritus but declined further treatment for the residual hyperpigmentation. This highlights the potential clinical impact of lichen planus pigmentosus, which, while often benign, can lead to lasting hyperpigmentation which may be cosmetically distressing. This illustrates the importance of recognizing potential rare dermatologic reactions to newer classes of medications like GLP-1 and GIP receptor agonists in order to provide early management of symptoms.

When starting a patient on GLP-1 and GIP receptor agonists, clinicians should be aware of its potential to cause lichen planus pigmentosus and that additional monitoring of dermatologic side effects may be necessary as additional cutaneous reactions may be underreported or misdiagnosed. Increasing our understanding of the potential association of these drugs with dermatologic side effects will improve patient counseling and allow for a more informed decision-making regarding their use. In conclusion, as drugs such as tirzepatide become more widespread it should be considered as a potential trigger for drug-induced lichen planus pigmentosus. With a high level of suspicion for dermatologic manifestations in individuals who develop new or worsening dermatologic symptoms after initiating this medication and when other more likely alternatives have been ruled out.

## Conflicts of interest

None disclosed.
